# Evaluation of FIB-4, NFS, APRI and Liver Function Tests as Predictors for SARS-CoV-2 Infection in the Elderly Population: A Matched Case-Control Analysis

**DOI:** 10.3390/jcm11175149

**Published:** 2022-08-31

**Authors:** Mirela Loredana Grigoras, Ioana Mihaela Citu, Cosmin Citu, Veronica Daniela Chiriac, Florin Gorun, Mihaela Codrina Levai, Diana Manolescu, Ovidiu Rosca, Felix Bratosin, Srivathsava Gurumurthy, Prima Hapsari Wulandari, Octavian Marius Cretu

**Affiliations:** 1 Department of Anatomy and Embryology, “Victor Babes” University of Medicine and Pharmacy Timisoara, Eftimie Murgu Square 2, 300041 Timisoara, Romania; 2 Department of Internal Medicine I, “Victor Babes” University of Medicine and Pharmacy Timisoara, Eftimie Murgu Square 2, 300041 Timisoara, Romania; 3 Department of Obstetrics and Gynecology, “Victor Babes” University of Medicine and Pharmacy Timisoara, Eftimie Murgu Square 2, 300041 Timisoara, Romania; 4 Research Center for Medical Communication, “Victor Babes” University of Medicine and Pharmacy Timisoara, Eftimie Murgu Square 2, 300041 Timisoara, Romania; 5 Department of Radiology, “Victor Babes” University of Medicine and Pharmacy Timisoara, Eftimie Murgu Square 2, 300041 Timisoara, Romania; 6 Methodological and Infectious Diseases Research Center, Department of Infectious Diseases, “Victor Babes” University of Medicine and Pharmacy Timisoara, 300041 Timisoara, Romania; 7 Mysore Medical College and Research Institute, Rajiv Gandhi University of Health Sciences, Irwin Road, Mysuru 570001, India; 8 Massachusetts General Hospital, Harvard Medical School, 55 Fruit St., Boston, MA 02114, USA; 9 Department of Surgery, Faculty of Medicine, “Victor Babes” University of Medicine and Pharmacy Timisoara, Eftimie Murgu Square 2, 300041 Timisoara, Romania

**Keywords:** SARS-CoV-2, COVID-19, FIB-4, NFS, APRI, inflammatory markers, liver function

## Abstract

Several investigations have revealed that COVID-19 causes a significant death rate due to acute respiratory distress syndrome, alterations in the quantity of ACE2 receptor expression, or the intensity of cytokine storm. Similarly, patients with hepatic impairment that are co-infected with SARS-CoV-2 are more likely to display upregulations of ACE2 receptors and cytokine storm overload, which exacerbates hepatic impairment, potentially increasing the death rate. Moreover, it is expected that the aging population develops a higher degree of hepatic fibrosis in association with other comorbid conditions that are likely to influence the course of COVID-19. Therefore, this research was developed to describe the differences in liver test parameters in elderly individuals with COVID-19 in relation to other inflammatory markers and outcomes. This current observational single-center research followed a case-control design of elderly patients hospitalized for SARS-CoV-2 infection. The research was conducted at a tertiary emergency hospital in western Romania during a two-year period. There were 632 patients included in the analysis that were split into two equal groups matched 1:1 based on gender and body mass index. Three hundred sixteen patients made the group of cases with COVID-19 patients older than 65 years, while the other half were the 316 patient controls with COVID-19 that were younger than 65 years old. Disease outcomes showed a higher prevalence of ICU admissions (22.8% vs. 12.7%, *p*-value < 0.001) and in-hospital mortality (17.1% vs. 8.9%, *p*-value = 0.002) in the group of cases. Specific and non-specific liver biomarkers were identified as risk factors for mortality in the elderly, such as ALP (OR = 1.26), LDH (OR = 1.68), AST (OR = 1.98), and ALT (OR = 2.34). Similarly, patients with APRI and NFS scores higher than 1.5 were, respectively, 2.69 times and, 3.05 times more likely to die from COVID-19, and patients with FIB-4 scores higher than 3.25 were 3.13 times more likely to die during hospitalization for SARS-CoV-2 infection. Our research indicates that abnormally increased liver biomarkers and high liver fibrosis scores are related to a worse prognosis in SARS-CoV-2 infected individuals.

## 1. Introduction

The World Health Organization (WHO) identified a cluster of respiratory illness cases with an unknown origin in Wuhan, China, in December 2019 [1]. The respiratory illness, which was subsequently called ‘Coronavirus Disease 2019’ (COVID-19), spread rapidly to neighboring states and has turned into a worldwide pandemic [2,3]. The condition is caused by the RNA virus “severe acute respiratory syndrome coronavirus 2” (SARS-CoV-2) and is spread mostly via respiratory droplets and intimate contact with an infected person [4,5]. As of April 2022, WHO has verified around 500 million cases of COVID-19 globally, with more than 6 million fatalities [6]. Patients with COVID-19 often have abnormal liver function, and some investigations have demonstrated that SARS-CoV-2 is related to liver dysfunction or damage [7]. There are almost 400,000 chronic liver disease patients in Romania [8], and doctors should be aware of the danger of liver involvement in COVID-19 patients, that several studies describe, particularly when the patient suffers from a pre-existent chronic liver condition [9,10,11].

SARS is mostly a lung infection, thus being named ‘Severe Acute Respiratory Syndrome’ and ‘SARS atypical pneumonia’. However, various organ dysfunctions have been reported in individuals, including gastrointestinal complaints, poor liver function, lymphadenopathy, and splenic atrophy [12]. However, most uncommon complications during and after COVID-19 have been reported in patients with multiple comorbidities, impaired immunity, and malignancies such as lung cancer [13,14]. These findings are indicative of extensive immunopathology or of SARS-coronavirus extrapulmonary dissemination and replication. Autopsies also suggest that the virus has infected various organs besides the liver [15]. The pathogenic alterations may be explained by either the virus’s direct cytotoxic impact on the host or by an immunological response caused by the virus. Certain findings indicate that COVID-19 may have an effect on other organs, including variable degrees of liver damage in infected individuals [16]. Recent research discovered that the SARS-CoV-2 virus binding to ACE 2 (ACE2) on cholangiocytes causes its malfunction, which may result in liver harm through an inflammatory response at the systemic level [17,18]. Several hospital-based investigations have documented liver damage in individuals with COVID-19 in the form of increased aspartate aminotransferase (AST) and alanine aminotransferase (ALT) values ranging from 14% to 53% above normal [19,20,21]. Additionally, the presence of modest microvesicular steatosis and lobular and portal activity in the liver biopsy specimens of a deceased COVID-19 patient revealed that SARS-CoV-2 was involved in liver damage [22].

Furthermore, it has been shown that older persons are more prone to get infected with the SARS-CoV-2 virus [23]. Early reports from China indicated a rise in the severity of sickness and death among persons aged 65 and older, and similar trends were seen in Europe, with mortality rates as high as 10% among adults aged 70, compared to 1% among young adults [24,25]. Compared to younger persons, elderly patients have a greater requirement for intensive care unit (ICU) admission and mechanical ventilation; hence, the SARS-CoV-2 infection increases the death risk among those aged 65 and older [26,27]. A subsequent meta-analysis supported these results, observing that almost half of older patients with COVID-19 have a severe infection, one in five are seriously sick, and one in ten will eventually die [28]. Moreover, older patients with a respiratory infection may appear with exhaustion, anorexia, and delirium in the absence of fever and productive cough, which may result in a delayed diagnosis and contribute to increased mortality in the elderly [29]. Among other clinical features and complications in this population, it was reported that their recovery from SARS-CoV-2 infection is prolonged, leading to the so-called “long COVID”, irreversible lung damage, and psychological complications from maladaptive stress [30,31,32].

With time passing, COVID-19 becomes a “mature” infection that becomes better understood by its molecular mechanisms and effects on the human body. The development of an efficient vaccine and a strong vaccination campaign among developed countries [33,34,35], with over 4.5 billion people vaccinated worldwide at the time of the study [36], allowed the populations at risk, such as the elderly, to become less vulnerable to COVID-19 [37,38]. However, the multitude of SARS-CoV-2 mutations that occurred allowed the virus to escape some immune mechanisms, determining infection with complications even in triple-vaccinated patients [39]. Therefore, this research aims to describe the differences in liver test parameters in elderly individuals with COVID-19 in relation to other inflammatory markers and outcomes.

## 2. Materials and Methods

### 2.1. Background, Design, and Ethics

This current observational single-center research followed a case-control design of elderly patients hospitalized for SARS-CoV-2 infection. The research was conducted at a tertiary emergency hospital in western Romania, where patients were hospitalized in the COVID-19 unit of the Timisoara Municipal Hospital. Data were collected between 1 March 2020 and 1 March 2022. The sample size and key features were identified via the use of a population-based administrative database of patients who attended the same clinic’s inpatient setting over the study period. Our comprehensive database included patient medical records that were protected by privacy laws and gathered with the patient’s permission. The patient’s demographics, medical history, laboratory profile, and in-hospital treatment were all included in this data. All patients’ baseline characteristics and procedures were recorded in the hospital database and in paper patient records inspected by certified clinicians participating in the current inquiry.

The Ethics Committee of the “Victor Babes” University of Medicine and Pharmacy in Timisoara, Romania, as well as the Ethics Committee of the Timisoara Municipal Hospital, accepted the study protocol. The institutions are governed by the provisions of Article 167 of Law No. 95/2006, Article 28 of Order 904/2006, and the EU Good Clinical Practice Directives 2005/28/EC, the International Conference on Harmonization of Technical Requirements for the Registration of Pharmaceuticals for Human Use (ICH), and the Declaration of Helsinki—Recommendations Guiding Mediation. On 23 December 2021, the present research was accepted with approval number I-32467.

### 2.2. Inclusion Criteria, Patient Characteristics, and Variables

The inclusion criteria were established for all patients over the age of 65, as a threshold for the age of retirement in Romania, as well as a threshold for old age as considered by multiple medical research studies [40,41]. Patients were included if they had a history of hospitalization in our department for SARS-CoV-2 infection, as determined by real-time polymerase chain reaction (RT-PCR). Exclusion criteria accounted for patients who suffered from cirrhosis, insufficient patient profiles in terms of imaging exams and laboratory data, as well as records without patient consent. The collection of data was performed by qualified doctors who volunteered to participate in this study, and the database information was validated against existing patient paper records. From the elderly population hospitalized in the COVID-19 department, we included a total of 316 cases of SARS-CoV-2 infection that were eligible for inclusion. The cohort of elderly patients with COVID-19 validated for study inclusion was compared with 316 adult patients younger than 65 years and a history of SARS-CoV-2 infection matched by gender and body mass index (BMI).

All individuals got a SARS-CoV-2 outpatient examination, having the infection validated by nasopharyngeal RT-PCR for SARS-CoV-2 RNA, in accordance with criteria at the time of the research. The infections were classified as mild, moderate, or severe COVID-19 based on clinical findings and computed tomography (CT) data. Therefore, all patients with pulmonary lesions were classified as mild (30 percent pulmonary damage), moderate (30–60 percent pulmonary damage), or severe (more than 60 percent of lung area damaged). A complete medical history, clinical examination, and other additional studies were assessed at the initial presentation. Representatives of the clinical teams collected anonymized laboratory data on all cases of COVID-19 diagnosed throughout the research period, including the following biological parameters: the red blood cell count, white blood cell count, hemoglobin, hematocrit, platelets, ferritin, erythrocyte sedimentation rate, c-reactive protein, fibrinogen, procalcitonin, d-dimers, interleukin-6, and creatinine. The liver studies comprised a fasting glucose check, alanine aminotransferase, aspartate aminotransferase, alkaline phosphatase, serum albumin, total proteins, total bilirubin, gamma glutamate transpeptidase, lactate dehydrogenase, prothrombin time, partial thromboplastin time, FIB-4, NFS, and APRI scores.

Other variables analyzed included the background characteristics of patients (age, body mass index, gender), area of residence, smoking status, history of an alcohol use disorder, the status of complete COVID-19 vaccination with three doses, comorbidities (malignancy, chronic lung disease, cardiovascular disease, cerebrovascular disease, diabetes mellitus, autoimmune disease, chronic kidney disease, digestive and liver disease), Charlson Comorbidity Index (CCI), oxygen supplementation, COVID-19 severity, intensive care unit admission, and in-hospital mortality. The Fibrosis-4 (FIB-4), nonalcoholic fatty liver disease (NAFLD), non-invasive fibrosis score (NFS), and AST to platelet ratio index (APRI) scores were reported as markers of liver fibrosis and clinical outcomes for the elderly admitted for SARS-CoV-2 infection and calculated as indicated by their validation studies [42,43,44] using the following formulas:

Fibrosis-4: [age (years) × AST (U/L)]/[platelet (×10^9^/L) × ALT (U/L)]; (1)

NAFLD fibrosis score: −1.675 + 0.037 × age (years) + 0.094 × body mass index (BMI) (kg/m^2^) + 1.13 × diabetes (yes = 1, no = 0) + 0.99 × AST [U/L]/ALT [U/L] − 0.013 × platelet (10^9^/L) − 0.66 × albumin (g/dL); (2)

APRI: [AST (U/L)/upper limit of normal × 100]/platelet (×10^9^/L) ratio; (3)

### 2.3. Statistical Analysis

The sample size was calculated for a confidence interval of 95%, margin of error 5% and a population proportion of approximately 20% of people older than 65 from the 19 million population size, according to the most recent statistics for age structure in Romania [45]. The sample size was considered adequate for a calculated number of 246 patients. For statistical analysis, MS EXCEL and IBM SPSS were used. Continuous variables were presented as the mean, standard deviation (SD), or median with interquartile range (IQR). Descriptive statistical analyses were done to obtain the means and standard deviations, and the Student’s *t*-test was used to estimate the *p*-value. To examine proportional differences, the Chi-square test was performed. The liver markers were included in a multivariate regression analysis that was adjusted for confounding factors (age, comorbidities, and COVID-19 vaccination status), with results expressed as odds ratio (OR) and confidence interval (CI). A *p*-value of 0.05 was determined to be statistically significant.

## 3. Results

### 3.1. Comparison of Baseline Characteristics

The selection process ended with the inclusion of 632 patients, where 316 made the group of cases with COVID-19 patients older than 65 years, while the other half were the 316 patient controls with COVID-19 that were younger than 65 years old and were matched 1:1 by gender and body mass index. After matching, there were 172 men in each group (54.4%). Table 1 represents the comparison of baseline characteristics between the two groups, identifying the mean age of 58.0 years of patient controls, compared with 71.4 years in the group of cases (*p*-value < 0.001). A statistically significant difference was observed between the cardiovascular and cerebrovascular comorbidities between the cases (respectively, 41.8% vs. 33.9%, *p*-value = 0.040) and controls (respectively, 15.5% vs. 7.6%, *p*-value = 0.001). Oxygen supplementation was required in a significantly higher proportion for the elderly patients, where only 3.8% did not require oxygen during admission, compared to 11.1% in the younger group of patients (*p*-value < 0.001). The higher use of oxygen supplementation is explained by a higher COVID-19 severity in the elderly, where 38.3% were severe cases, compared to 28.2% in the control group (*p*-value = 0.016). Also, disease outcomes showed a higher prevalence of ICU admissions (22.8% vs. 12.7%, *p*-value < 0.001) and in-hospital mortality (17.1% vs. 8.9%, *p*-value = 0.002) in the group of cases.

### 3.2. Laboratory Profile Analysis

The laboratory profile analysis described in Table 2 compares the values outside the normal range of biological parameters during hospitalization between the cases and controls. It was observed that a significantly higher proportion of the elderly patients had their serum markers outside the range of normal values. The red blood cell count, hemoglobin, and hematocrit were significantly decreased, while the white blood cells, ESR, procalcitonin, D-dimers, and creatinine were statistically significantly higher than in the group of controls.

**Figure 1:**
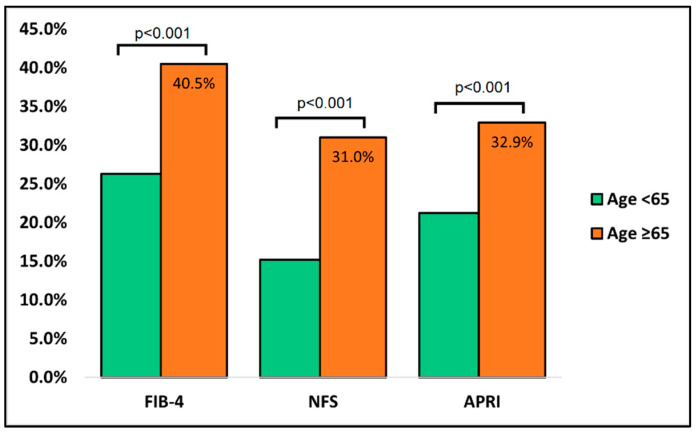
Comparison of liver fibrosis scores between cases and controls at admission.

Table 3 describes the comparison of liver studies that are routinely performed at admission between cases and controls. It was observed that patients older than 65 had significantly more alterations in liver serum markers and liver fibrosis scores. ALT and AST were significantly more elevated in the older patient group, where approximately 57 percent of all patients had alterations outside the normal range, compared with 41 percent in the younger patient group (*p*-value < 0.001). LDH was also significantly higher in the cases group (38.0% vs. 27.8%, *p*-value = 0.006). After calculating the liver fibrosis scores, it was determined that FIB-4, NFS, and APRI had significantly higher proportions of abnormal values in the older patients (Figure 1).

**Figure 2:**
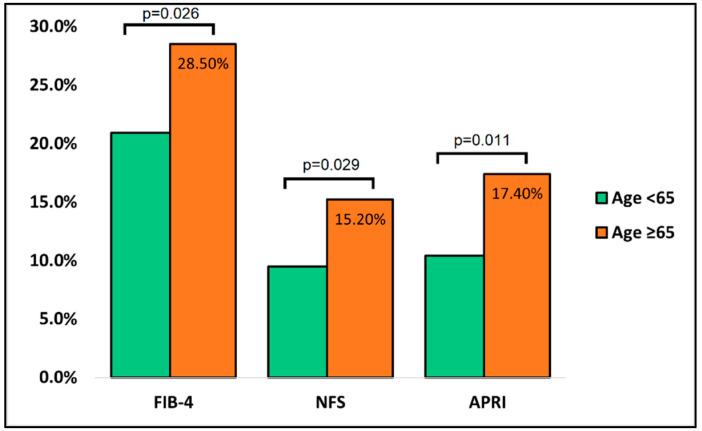
Comparison of liver fibrosis scores between cases and controls at discharge.

The comparison of liver studies at discharge between the surviving cases and controls is presented in Table 4. A total of 288 patients in the group of controls were analyzed, compared with 262 that survived from the group of cases. Similarly, with the admission findings, the ALT, AST, and ALP showed significantly higher alterations in patients older than 65 years, as well as in the number of total proteins (22.5% vs. 15.2%, *p*-value = 0.019). The FIB-4 score was outside the normal range of 1.45–3.25 in the cases group in 28.5% of measurements, compared with 20.9% of measurements in the controls (*p*-value = 0.026). Similar findings were observed in NFS and APRI fibrosis scores (*p*-value = 0.029 and *p*-value = 0.011, respectively), as described in Figure 2.

**Figure 3:**
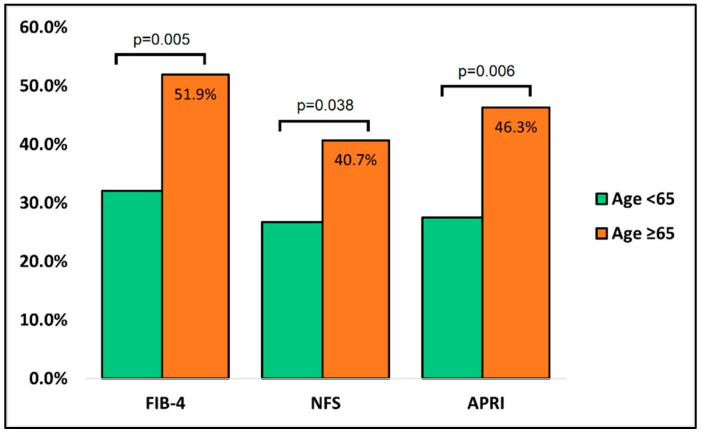
Comparison of liver fibrosis scores between survivors and deceased cases at admission.

The comparison of liver studies at admission between the surviving and deceased cases of older patients is presented in Table 5, having a total of 262 patients who survived and 54 deaths. In contrast with the comparison of liver studies between cases and controls at admission, the discrepancy of findings was higher between deceased and survivors. Statistically significant differences were observed among proportions of all serum liver markers, except for total bilirubin. FIB-4, NFS, and APRI scores were significantly elevated at hospital admission in patients who died. As seen in Figure 3, there were 28 (51.9%) with FIB-4 scores higher than 3.25 among the deceased, compared to 26.7% in the group of survivors (*p*-value = 0.005). Similarly, the NFS score below the normal range was in 40.7% of deceased patients, as opposed to survivors, where 26.7% were below the normal range (*p*-value = 0.038). Lastly, the APRI score was abnormal in 46.3% of deceased patients at admission, compared with only 27.5% of survivors (*p*-value = 0.006).

### 3.3. Risk Factor Analysis

**Figure 4:**
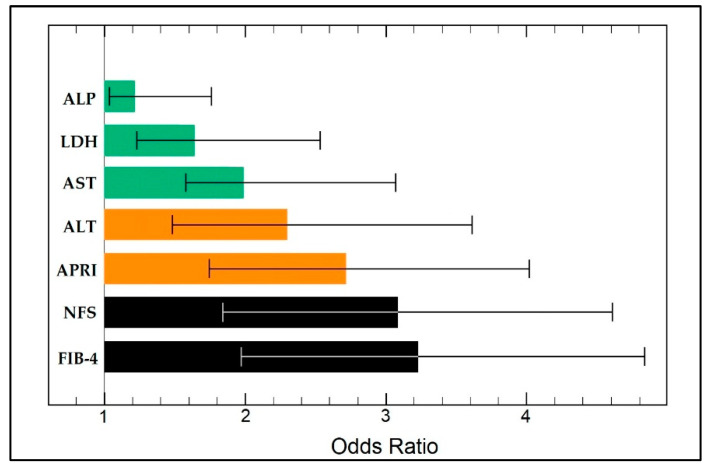
Multivariate risk factor analysis for mortality in the elderly (≥65 years old) admitted for SARS-CoV-2 infection.

The risk factor analysis was performed to determine the independent predictors for mortality in the elderly admitted with COVID-19 based on specific and non-specific liver biomarkers. As presented in Table 6 and Figure 4, in ascending order of odds ratio, it was determined that ALP (OR = 1.26), LDH (OR = 1.68), AST (OR = 1.98), and ALT (OR = 2.34) were statistically significant independent risk factors for mortality. The association of APRI, NFS, and FIB-4 scores with mortality in the elderly with COVID-19 was highly significant for all three scores (*p*-value < 0.001). Therefore, patients with APRI and NFS scores higher than 1.5 were 2.69 times and 3.05 times, respectively, more likely to die from COVID-19 infection than the control patients (CI = 1.52–3.66 CI = 1.83–4.61, respectively). Similarly, patients with FIB-4 scores higher than 3.25 were 3.13 times more likely to die during hospitalization for SARS-CoV-2 infection (CI = 1.95–4.86).

## 4. Discussion

### 4.1. Important Findings

The current study managed to identify significant differences between the elderly and the younger controls in specific and non-specific liver studies that can be routinely performed on admission for SARS-CoV-2 infection. The APRI, NFS, and FIB-4 liver fibrosis scores outperformed the other serum biomarkers that were monitored at admission and discharge. Similar findings were reported in the analysis of FIB-4 in a Romanian study [46]. It was observed that, in patients with COVID-19, more than 70 percent had abnormal liver biomarkers, and mortality was independently linked with severe FIB-4 over the 3.25 threshold and high alanine aminotransferase levels. In addition, the authors determined the survival probability based on abnormal liver biochemistry on arrival and severe FIB-4 scores, with an area under the receiver operating characteristic of 0.73 for predicting survival, demonstrating that fibrosis scores like FIB-4 could aid clinicians in categorizing patients—in terms of prognosis—and approaching them accordingly, and perhaps even prioritizing them for vaccination because they represent a group with a poorer prognosis.

According to these findings, a systematic review of the relationship between liver fibrosis scores and outcomes in SARS-CoV-2-infected patients reveals that high liver fibrosis scores are linked with a worse prognosis in COVID-19-infected individuals [47]. Patients with COVID-19 upon admission, particularly those with concurrent chronic liver disorders, may benefit from an evaluation of liver fibrosis scores to identify individuals at high risk of developing severe COVID-19 cases and poor outcomes. Comparable with our results, the NFS score has also been observed in the literature [48] as a significant predictor for negative outcomes in COVID-19 patients, although tightly correlated with the presence of non-alcoholic fatty liver disease (NAFLD). More than forty percent of NAFLD patients with COVID-19 had liver fibrosis according to the NFS, and the combination of NAFLD and elevated NFS constituted an independent risk factor for severe disease in those admitted to hospital for SARS-CoV-2 infection [49]. However, patients in our cohort were not selected or stratified by the presence of NAFLD, while the fibrosis score calculated abnormal values in many of the patients with negative outcomes.

Several alternative pathways have been postulated to explain how certain degrees of liver fibrosis can lead to a more severe SARS-CoV-2 infection in NAFLD and also non-NAFLD patients, but the specific pathophysiology is still unclear. The systemic inflammatory response syndrome, particularly the cytokine storm, is one of the key pathophysiologic processes behind the development of severe disease in COVID-19 [50]. The presence of fibrosis may worsen the virus-induced cytokine storm and add to the severity of COVID-19 by the production of proinflammatory cytokines from the liver [51]. However, further research is required to investigate the pathways through which advanced liver fibrosis contributes to the COVID-19 pathological mechanisms.

Several variables, such as the healthcare system policies, patient characteristics, and prevalence of diagnostic testing, may account for the observed disparities in this research. Comorbidities such as hypertension, diabetes, and obesity have been demonstrated to be related to greater COVID-19 mortality, and they rise continuously with age, which might be another plausible reason for the observed increase in death in older patients. While illness mortality is greater in the elderly for other disorders, such as cardiovascular disease, this may explain the increased susceptibility to infection and the disproportionately high death rate linked with COVID-19 in older individuals [52].

### 4.2. Strengths and Limitations

One of the current study’s strengths is that, based on previous data, it was decided that cases and controls should be matched by gender since men have higher odds for mortality after SARS-CoV-2 infection than women, as determined by one meta-analysis [46]. Also, the body mass index was another variable based on which matching was performed to avoid a multitude of comorbidities tightly associated with it as a bias factor [53]. Complete COVID-19 vaccination status was a variable of interest for adjusting risk factors, even though this early study did not identify many hospitalized patients that received all three vaccine doses. Considering that nowadays the COVID-19 vaccination coverage in Western European countries and United States reaches up to an average of 70% [54,55], our findings might not be reproducible for these populations with high vaccination coverage. Another limitation is that COVID-19 fatalities can be underestimated to the degree that patients died from COVID-19 without being diagnosed as SARS-CoV-2-positive. Considering this underestimation of COVID-19 mortality is significantly bigger among the elderly, the study might have underestimated the size of the relative death rate among individuals of this age group, which can be considered an important limitation. Another drawback is the retrospective cohort design since the assessment of risk variables and outcomes through a manual search of patient records and personal files is likely to be less precise and consistent than in prospective cohort research.

## 5. Conclusions

While decreasing the stresses on the health service and reviving the economy, it will be crucial that future decisions take into consideration the demographics of the population, notably the proportion of those 65 and older in specific areas or towns where nursing homes are situated. Monitoring the dynamics and interconnections of the aging population may give more insight into how to safeguard the more vulnerable older population.

## Figures and Tables

**Table 1:** Comparison of baseline characteristics between cases and controls.

Variables *	Age < 65 (*n* = 316)	Age ≥ 65 (*n* = 316)	*p*-Value
**Background data**			
Age, years (mean ± SD)	58.0 ± 11.8	71.4 ± 9.2	<0.001
BMI, kg/m^2^ (mean ± SD)	24.4 ± 4.1	24.8 ± 4.0	0.214
Gender (men)	172 (54.4%)	172 (54.4%)	1
Area of residence (urban)	187 (59.2%)	169 (53.5%)	0.148
Smoking	114 (36.1%)	95 (30.1%)	0.108
Chronic alcohol use	24 (7.6%)	29 (9.2%)	0.473
Complete COVID-19 Vaccination	7 (2.2%)	13 (4.1%)	0.172
Hepatitis B vaccine	11 (3.5%)	6 (1.9%)	0.218
**Comorbidities**			
Malignancy	18 (5.7%)	25 (7.9%)	0.268
Chronic lung disease	28 (8.9%)	39 (12.3%)	0.155
Cardiovascular disease	107 (33.9%)	132 (41.8%)	0.040
Cerebrovascular disease	24 (7.6%)	49 (15.5%)	0.001
Diabetes mellitus	41 (13.0%)	48 (15.2%)	0.423
Autoimmune disease	13 (4.1%)	15 (4.7%)	0.699
Chronic kidney disease	16 (5.1%)	21 (6.6%)	0.396
Digestive and liver disease **	23 (7.3%)	30 (9.5%)	0.315
CCI score (≥2)	76 (24.1%)	107 (33.9%)	0.006
**Oxygen supplementation**			<0.001
No supplementation	35 (11.1%)	12 (3.8%)	
Non-invasive ventilation	241 (76.3%)	248 (78.5%)	
Invasive ventilation	40 (12.7%)	56 (17.7%)	
**COVID-19 severity**			0.016
Mild	106 (33.5%)	82 (25.9%)	
Moderate	121 (38.3%)	113 (35.8%)	
Severe	89 (28.2%)	121 (38.3%)	
**Disease outcomes**			
Days of hospitalization (mean ± SD)	11 ± 6.6	19 ± 8.3	<0.001
ICU admission	40 (12.7%)	72 (22.8%)	<0.001
In-hospital mortality	28 (8.9%)	54 (17.1%)	0.002

* Data reported as n (%) and calculated using Chi-square test and Fisher’s exact unless specified differently; ** Liver disease including fatty liver disease, hepatitis B and C infection, drug-induced liver disease; BMI—body mass index; ICU—intensive care unit; CCI—Charlson Comorbidity Index.

**Table 2:** Comparison of biological parameters during hospitalization between cases and controls.

Variables *	Normal Range	Age < 65 (*n* = 316)	Age ≥ 65 (*n* = 316)	*p*-Value
RBC (millions/mm^3^)	4.35–5.65	61 (19.3%)	108 (34.2%)	<0.001
WBC (thousands/mm^3^)	4.5–11.0	72 (22.8%)	121 (38.3%)	<0.001
Hemoglobin (g/dL)	13.0–17.0	54 (17.1%)	83 (26.3%)	0.005
Hematocrit (%)	36–48	39 (12.3%)	67 (21.2%)	0.002
Platelets (thousands/mm^3^)	150–450	41 (13.0%)	53 (16.8%)	0.179
Ferritin (ng/mL)	20–250	48 (15.2%)	66 (20.9%)	0.062
ESR (mm/h)	0–22	124 (39.2%)	149 (47.2%)	0.044
CRP (mg/L)	0–10	145 (45.9%)	166 (52.5%)	0.094
Fibrinogen (g/L)	2–4	169 (53.5%)	180 (57.0%)	0.378
Procalcitonin (ug/L)	0–0.25	53 (16.8%)	92 (29.1%)	<0.001
D-dimers (ng/mL)	<250	27 (8.5%)	44 (13.9%)	0.032
IL-6 (pg/mL)	0–16	52 (16.5%)	65 (20.6%)	0.183
Creatinine (µmol/L)	0.74–1.35	30 (9.5%)	68 (21.5%)	<0.001

* Data reported as % outside the normal range and calculated using Chi-square test and Fisher’s exact unless specified differently; RBC—red blood cells; WBC—white blood cells; ESR—erythrocyte sedimentation rate; CRP—C-reactive protein; IL-6—interleukin 6.

**Table 3:** Comparison of liver studies at admission between cases and controls.

Variables *	Normal Range	Age < 65 (*n* = 316)	Age ≥ 65 (*n* = 316)	*p*-Value
Fasting glucose (mmol/L)	60–125	39.2%	48.4%	0.020
ALT (U/L)	7–35	41.5%	56.3%	<0.001
AST (U/L)	10–40	40.2%	57.6%	<0.001
ALP (U/L)	40–130	34.5%	52.8%	<0.001
Serum albumin (g/dL)	3.4–5.4	31.3%	36.1%	0.206
Total proteins (g/dL)	6.0–8.3	26.3%	30.4%	0.251
Total bilirubin (g/dL)	0.3–1.2	20.6%	25.6%	0.131
GGT (U/L)	0–30	22.8%	27.2%	0.198
LDH (U/L)	140–280	27.8%	38.0%	0.006
PT (seconds)	11.0–13.5	28.8%	34.5%	0.123
APTT (seconds)	30–40	27.2%	32.3%	0.163
FIB-4	1.45–3.25	26.3%	40.5%	<0.001
NFS	<−1.5	15.2%	31.0%	<0.001
APRI	0.5–1.5	21.2%	32.9%	<0.001

* Data reported as % outside the normal range and calculated using Chi-square test and Fisher’s exact unless specified differently; ALT—alanine aminotransferase; AST—aspartate aminotransferase; ALP—alkaline phosphatase; GGT—gamma glutamyl transpeptidase; LDH—lactate dehydrogenase; PT—prothrombin time; APTT—activated partial thromboplastin clotting time; FIB-4—Fibrosis-4 score; NFS—nonalcoholic fatty liver disease fibrosis score; APRI—AST to platelet ratio index.

**Table 4:** Comparison of liver studies at discharge between cases and controls.

Variables *	Normal Range	Age < 65 (*n* = 288)	Age ≥ 65 (*n* = 262)	*p*-Value
Fasting glucose (mmol/L)	60–125	31.3%	36.7%	0.153
ALT (U/L)	7–35	27.8%	35.1%	0.048
AST (U/L)	10–40	29.1%	37.7%	0.022
ALP (U/L)	40–130	22.8%	30.4%	0.030
Serum albumin (g/dL)	3.4–5.4	21.2%	25.9%	0.159
Total proteins (g/dL)	6.0–8.3	15.2%	22.5%	0.019
Total bilirubin (g/dL)	0.3–1.2	12.3%	16.8%	0.363
GGT (U/L)	0–30	15.2%	20.9%	0.062
LDH (U/L)	140–280	13.0%	16.8%	0.179
PT (seconds)	11.0–13.5	17.1%	21.8%	0.131
APTT (seconds)	30–40	16.8%	20.6%	0.220
FIB-4	1.45–3.25	20.9%	28.5%	0.026
NFS	<−1.5	9.5%	15.2%	0.029
APRI	0.5–1.5	10.4%	17.4%	0.011

* Data reported as % outside the normal range and calculated using Chi-square test and Fisher’s exact unless specified differently; ALT—alanine aminotransferase; AST—aspartate aminotransferase; ALP—alkaline phosphatase; GGT—gamma glutamyl transpeptidase; LDH—lactate dehydrogenase; PT—prothrombin time; APTT—activated partial thromboplastin clotting time; FIB-4—Fibrosis-4 score; NFS—nonalcoholic fatty liver disease fibrosis score; APRI—AST to platelet ratio index.

**Table 5:** Comparison of liver studies at admission between survivors and deceased elderly with COVID-19.

Variables *	Normal Range	Survivors (*n* = 262)	Deceased (*n* = 54)	*p*-Value
Fasting glucose (mmol/L)	60–125	96 (36.6%)	29 (53.7%)	0.019
ALT (U/L)	7–35	68 (32.4%)	31 (57.4%)	<0.001
AST (U/L)	10–40	97 (37.0%)	36 (66.7%)	<0.001
ALP (U/L)	40–130	101 (38.5%)	30 (55.6%)	0.020
Serum albumin (g/dL)	3.4–5.4	64 (24.4%)	25 (46.3%)	0.001
Total proteins (g/dL)	6.0–8.3	67 (25.6%)	23 (42.6%)	0.011
Total bilirubin (g/dL)	0.3–1.2	60 (22.9%)	18 (33.3%)	0.105
GGT (U/L)	0–30	63 (24.0%)	20 (37.0%)	0.048
LDH (U/L)	140–280	75 (28.6%)	24 (44.4%)	0.022
PT (seconds)	11.0–13.5	71 (27.1%)	21 (38.9%)	0.082
APTT (seconds)	30–40	63 (24.0%)	19 (35.2%)	0.089
FIB-4	1.45–3.25	84 (32.1%)	28 (51.9%)	0.005
NFS	<−1.5	70 (26.7%)	22 (40.7%)	0.038
APRI	0.5–1.5	72 (27.5%)	25 (46.3%)	0.006

* Data reported as n (%) outside the normal range and calculated using Chi-square test and Fisher’s exact unless specified differently; ALT—alanine aminotransferase; AST—aspartate aminotransferase; ALP—alkaline phosphatase; GGT—gamma glutamyl transpeptidase; LDH—lactate dehydrogenase; PT—prothrombin time; APTT—activated partial thromboplastin clotting time; FIB-4—Fibrosis-4 score; NFS—nonalcoholic fatty liver disease fibrosis score; APRI—AST to platelet ratio index.

**Table 6:** Associations between significant liver markers and fibrosis scores at admission with mortality in the elderly patients with COVID-19.

Risk Factors	OR	95% CI	*p*-Value
ALP (U/L)	1.26	1.03–1.84	0.033
LDH (U/L)	1.68	1.22–2.97	0.001
AST (U/L)	1.98	1.49–3.15	0.001
ALT (U/L)	2.34	1.52–3.66	<0.001
APRI > 1.5	2.69	1.65–4.07	<0.001
NFS > 1.5	3.05	1.83–4.61	<0.001
FIB-4 > 3.25	3.13	1.95–4.86	<0.001

FIB-4—Fibrosis-4 score; NFS—nonalcoholic fatty liver disease fibrosis score; APRI—AST to platelet ratio index; ALT—alanine aminotransferase; AST—aspartate aminotransferase; ALP—alkaline phosphatase; LDH—lactate dehydrogenase.

## Data Availability

The data presented in this study are available on request from the corresponding author.
